# Remotely Sensed Data Fusion for Spatiotemporal Geostatistical Analysis of Forest Fire Hazard

**DOI:** 10.3390/s20175014

**Published:** 2020-09-03

**Authors:** Stavros Sakellariou, Pedro Cabral, Mário Caetano, Filiberto Pla, Marco Painho, Olga Christopoulou, Athanassios Sfougaris, Nicolas Dalezios, Christos Vasilakos

**Affiliations:** 1 NOVA Information Management School, Universidade Nova de Lisboa, 1070-312 Lisbon, Portugal; stasakel@gmail.com (S.S.); pcabral@novaims.unl.pt (P.C.); mario@novaims.unl.pt (M.C.); painho@novaims.unl.pt (M.P.); 2 Department of Planning and Regional Development, University of Thessaly, 38334 Volos, Greece; ochris@uth.gr; 3 Department of Agriculture Crop Production and Rural Environment, University of Thessaly, 38446 Volos, Greece; asfoug@agr.uth.gr; 4 Institute of New Imaging Technologies (INIT), Universitat Jaume I (UJI), 12071 Castellón, Spain; filiberto.pla@lsi.uji.es; 5 Department of Civil Engineering, University of Thessaly, 38334 Volos, Greece; 6 Department of Geography, University of the Aegean, University Hill, 81100 Mytilene, Greece; chvas@aegean.gr

**Keywords:** forest fire hazard, Analytical Hierarchy Process, fuzzy logic, spatiotemporal analysis, spatial variability, remote sensing

## Abstract

Forest fires are a natural phenomenon which might have severe implications on natural and anthropogenic ecosystems. Future projections predict that, under a climate change environment, the fire season would be lengthier with higher levels of droughts, leading to higher fire severity. The main aim of this paper is to perform a spatiotemporal analysis and explore the variability of fire hazard in a small Greek island, Skiathos (a prototype case of fragile environment) where the land uses mixture is very high. First, a comparative assessment of two robust modeling techniques was examined, namely, the Analytical Hierarchy Process (AHP) knowledge-based and the fuzzy logic AHP to estimate the fire hazard in a timeframe of 20 years (1996–2016). The former technique was proven more representative after the comparative assessment with the real fire perimeters recorded on the island (1984–2016). Next, we explored the spatiotemporal dynamics of fire hazard, highlighting the risk changes in space and time through the individual and collective contribution of the most significant factors (topography, vegetation features, anthropogenic influence). The fire hazard changes were not dramatic, however, some changes have been observed in the southwestern and northern part of the island. The geostatistical analysis revealed a significant clustering process of high-risk values in the southwestern and northern part of the study area, whereas some clusters of low-risk values have been located in the northern territory. The degree of spatial autocorrelation tends to be greater for 1996 rather than for 2016, indicating the potential higher transmission of fires at the most susceptible regions in the past. The knowledge of long-term fire hazard dynamics, based on multiple types of remotely sensed data, may provide the fire and land managers with valuable fire prevention and land use planning tools.

## 1. Introduction

Forest fires constitute a natural phenomenon and potentially a natural hazard which might have severe implications on natural and anthropogenic ecosystems [1,2,3,4]. Inter-annual fire statistics may provide a clear enough picture of fire regimes. In the USA, the annual number of fire events is 74,208, burning on average 2,129,203 ha from 1985 to 2018 [5]. For the same timeframe, the greatest annual amount of suppression cost required was more than $3 billion [6], without considering any human loss. In Europe, the five most fire-prone countries are located in the Mediterranean basin (Portugal, Spain, France, Italy, Greece). In this territory, the annual average number of fire events ranges from 1432 (Greece) to 18,025 (Portugal), burning from 24,200 (France) to 158,834 (Spain) ha each year for a timeframe of 39 years (1980–2018) [7]. Future projections predict that, under a climate change environment, the fire season (especially in Southern Europe) will be lengthier, with higher levels of droughts leading to higher fire severity [8]. These findings highlight the necessity of fire prevention measures in order to tackle any destructive consequences in the natural and socioeconomic web of any region.

Fire hazard/risk modeling itself constitutes a complex and multifaceted process since natural phenomena are characterized by a high degree of uncertainties [9]. Many researchers have developed fire hazard maps focusing on specific aspects that may heavily affect forest fires’ ignition and behavior. Eugenio et al. [10] estimated the fire hazard considering surface features (topography), meteorological factors, and the influence of human presence through linear entities (road network), as well as the potential flammability of territory through the nature of land cover types. In the same context, Sivrikaya et al. [11] paid special attention to the influence of forest vegetation features in terms of fire hazard, along with the contribution of geomorphology and artificial structures’ proximity. Vadrevu et al. [12] enriched their fire risk models incorporating fire behavior indices such as the amount of energy released during the burning process and biomass density, as well as other socioeconomic factors (literacy rate and population density, employment rate in primary sector of economy, etc.) that may potentially affect fire ignition. Amalina et al. [13] developed a susceptibility map focusing on the land use types, the meteorological conditions (through vegetation/climatological indices and surface temperature), and the proximity to natural and anthropogenic structures and entities (e.g., water bodies, crop types, road and human settlement network, etc.). Similarly, Sakellariou et al. [14] explored the distinct effect of natural and anthropogenic factors to fire hazard with emphasis on specific measures to alleviate their influence. Analytical Hierarchy Process (AHP) and fuzzy logic modeling have been widely applied to numerous fields in order to reduce the subjectivity and uncertainty of complex phenomena like forest fires vulnerability [12,15,16,17]. 

Other authors primarily supported their research with remote sensing data and techniques. Pradhan et al. [18] created a susceptibility map based on the interrelation of fire events (derived from National Oceanic and Atmospheric Administration–NOAA- satellites) and each contributing factor, such as the Normalized Difference Vegetation Index (NDVI), topographic (e.g., soil features, slope, aspect), and meteorological variables. Hence, based on the frequency ratio technique, they estimated the corresponding weights for all variables and developed the final risk map. On the other hand, Gabban et al. [19] estimated the fire risk for the Mediterranean countries through the development of an index that is reliant on the temporal evolution of NDVI values (derived from NOAA satellites), underlying the high correlation between this dynamic index and the number of fire events. Pourghasemi [20] compared two weighting schemes, namely, the evidential belief function and the binary logistic regression, of all the involved factors (topographic and meteorological factors, proximity, soil features, land use, NDVI, etc.) in order to estimate the fire risk, concluding that the latter technique was more effective.

Islands can be considered quite susceptible environments where any critical change may drastically affect every aspect of those territories, from the natural and cultural environment to socioeconomic life and cohesion. The island of Skiathos in Greece constitutes a unique case due to the fact that it is a highly touristic island and preserves forests and landscapes of extraordinary beauty. Therefore, this natural asset must be preserved and protected from one of the most damaging factors, namely, forest fires.

Hence, the main aim of the paper is to present a “proof of concept” for an integrated analysis of fire hazard through the combination of spatiotemporal and geostatistical assessment of fire hazard evolution using time-series data (1996–2016) from multiple remotely sensed sources (e.g., spectral data for vegetation status and moisture, surface geospatial data retrieved from airborne and satellite images such as relief, land cover, human constructions, etc.). This type of analysis would allow us to assimilate the fire hazard dynamics (under the threat of climate change) and develop the appropriate preventative measures in the most vulnerable regions. In the same context, we would be able to monitor the transformation of any high-risk clusters (through spatial autocorrelation) that could provoke severe fire phenomena affecting the nearby assets as well as the degree of heterogeneity (through semivariogram modeling) of fire hazard (translated as the ease of fire transmissibility) across the entire island through time. The interplay of the adopted techniques and sources allowed us to tackle serious data gaps due to the extremely spatial scale of the study area. Otherwise, the thorough fire hazard analysis of this vulnerable region would be impossible.

## 2. Materials and Methods

### 2.1. Study Area

**Figure 1:**
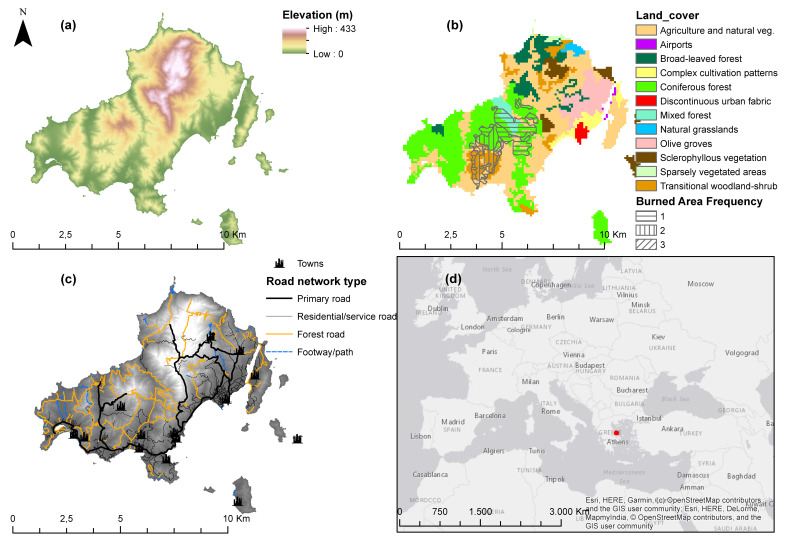
(**a**) Elevation, (**b**) land cover and burned areas, (**c**) spatial distribution of road and human settlements network, and (**d**) geographical position of Skiathos Island.

The study area of the paper is a small Greek island, Skiathos. The island of Skiathos is situated in the central area of Greece (Figure 1). The geographic coordinates of the study domain are: 39°10′ N 23°29′ E. The highest altitude of the study area is 433 m. The island is mainly occupied by lowland areas, followed by relatively small regions with higher altitudes in the north (Figure 1a). The total area amounts to 4887.7 ha, whereas the length of the coast is 44 km.

Concerning the land cover data, according to the Corine Land Cover (CLC) [21], 53.9% of the entire island is covered by different types of forests, 44.9% of the study area is occupied by agricultural fields, whereas only 1.1% of the island is occupied by artificial structures (land.copernicus.eu 2017). Consequently, the predominant land cover types favor forest fire ignition and extended spread, since the combination of coniferous and mixed forests along with the transitional woodland-shrub occupy more than 40% of the entire island (Figure 1b). 

Skiathos island has faced four intense forest fire events in almost twenty years, from 1984 to 2016. The most affected land covers consisted of transitional woodland-shrub (340.1 ha: 51% of the total burned area), coniferous forests (181.7 ha: 27.3% of the total burned area), and other territory (144.9 ha: 21.7% of the total burned area) [22]. Even though the characteristics of the fire regime cannot be determined in such a small spatial scale, we can have an indication about the fire conditions in the island. In order to understand the significance of adopting efficient fire prevention measures, it should be emphasized that in the above timeframe, almost 710 ha of burned area have been recorded, which is equivalent of almost 15% of the entire island (Figure 1b).

The population of Skiathos remained almost stable (+1.1%) in the last ten years, reaching 6088 people according to the last inventory of 2011 [23]. However, the incoming international tourists for 2017 were more than 182,000, which means 30 times greater than the permanent local population [24]. The human settlements are mainly concentrated on the coastal and lowland territories, whereas the road network is dense enough (Figure 1c). The spatial configuration of these anthropogenic structures may heavily affect the spatial patterns of fire ignitions.

Due to the extremely small spatial scale of the study area, there is a great shortage of historic climatic data. Based on the most recent weather data derived from the closest station (2015–2017), we can observe that the hottest months are July (average temperature: 28 °C, highest temperature: 38.7 °C) and August (average temperature: 27.9 °C, highest temperature: 36.7 °C). In addition, the lowest minimum relative humidity has been observed in August (42.6%), whereas the lowest level of precipitation has also been noted in August (9.6 mm) [25]. Therefore, the riskiest month is August, when high temperatures are accompanied with a marginal level of rainfall and the lowest level of moisture, a fact that could lead to a more severe forest fires phenomena [26].

### 2.2. Data and Pre-Processing

In order to estimate the fire hazard as reliably as possible, we retrieved and edited a series of different types of remotely sensed data that could potentially affect fire ignition and behavior. Thus, three types of data have been involved. Firstly, the Digital Elevation Model (DEM) has been used to calculate the elevation, slope, and aspect of the study area. This type of data was derived from the National Cadastre Agency [27] with a spatial resolution of 5 m. The DEM was produced by aerial images and photogrammetry techniques applied by the responsible agency). Topography is considered stable, since it is highly improbable to be changed in a timeframe of 20 years. Next, the land use data was retrieved from Copernicus Land Monitoring Service. Specifically, the land uses data for 1990 was retrieved from Landsat 5 (integrating Multispectral Scanner and the Thematic Mapper instruments), whereas the same data for 2012 was derived from a combination of remotely sensed sources, such as the Indian Remote-Sensing Satellite P6 Linear Imaging Self-Scanning Sensor (LISS III) and the RapidEye imagery [21]. However, the minimum mapping unit amounts to 25 ha, ignoring a significant number of artificial structures that have been created across the territory of one of the most touristic islands in Greece. To this end, a refinement process has been adopted. We digitized the artificial structures using orthophotos (aerial images) with a spatial resolution of 0.5 m for 1996 [27], and Google Earth for 2016. Thus, the land uses data of 1990 and 2012 have been enriched and refined including most of the structures present in 1996 and 2016, respectively. Similar work has been done for the road network. The road network has been digitized for 1996 [28], while the respective database for 2016 has been retrieved by Geofabrik [29]. The inhabited regions have been extracted through the refinement process of land uses for both years. 

Due to the shortage of coherent meteorological and fuels’ data, remote sensing indices have been used as proxy for fuels’ (vegetation) state and moisture data. Thus, we retrieved the two images for 1996 and 2016 in the same month (August). The former image was retrieved from the Landsat Thematic Mapper sensor (installed on Landsat 5), providing an image with six spectral bands and 30 m spatial resolution. The latter image was derived from the Operational Land Imager sensor (installed on Landsat 8), providing an image with nine spectral bands (Coastal, Blue, Green, Red, Near Infrared, Short-wavelength infrared 1, Short-wavelength infrared 2, Pan, Cirrus) and 30 m spatial resolution, ignoring the images coming from the Thermal Infrared Sensor. The selection of both images in August was conducted for two reasons: First, we had to retain the same phenology properties of vegetation for both reference years. Second, August is considered the hottest month in Greece with the least moisture levels, a fact that usually triggers the most intense and catastrophic forest fire events. Images without cloud interference have been selected [30,31]. Afterwards, a radiometric calibration of the involved bands has been conducted. Firstly, we transformed the Digital Number of each band to radiance through the Gain and Bias Method [32]. Finally, we had to transform the radiance to Top of Atmosphere Reflectance [32]. After all these preprocessing steps, we calculated the NDVI [33] and Normalized Difference Moisture Index (NDMI) [34]. 

Table 1 classifies the types of data used in forest fire hazard modeling, providing more specific details, such as the spatial resolution, the purpose, and the corresponding source for each category of geospatial data.

ArcGIS 10.4.1 [35] has been used for the geo-processing and geostatistical analysis. The involvement of all these types of geospatial data and the respective processes in such a small spatial scale constitute a novel approach handling multiple types of data that could optimize the final result. The final pixel size of the fire hazard maps is 30 m, which is identical with two of the most critical factors that are subject to change in the long-run (i.e., NDVI and NDMI). 

### 2.3. Methodology

**Figure 2:**
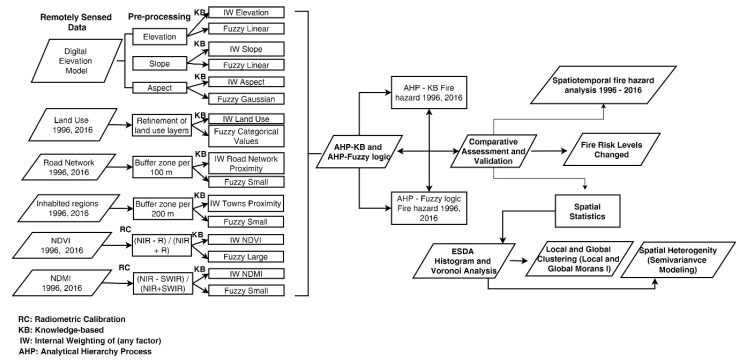
Flowchart of the spatiotemporal analysis and variability of fire hazard.

The methodology of the study consists of three parts. The first is related with the comparative analysis and assessment of two robust fire hazard estimation techniques, namely, the AHP-knowledge-based and AHP-fuzzy logic modeling of the most contributing factors. Next, a validation approach would allow the selection of the most representative technique based on real fire history data. Finally, a spatiotemporal and geostatistical analysis of the selected model is conducted in a timeframe of 20 years (1996–2016) in order to reveal the underlying spatial patterns of fire hazard. Figure 2 summarizes the flowchart of the adopted methodology.

#### 2.3.1. Classification of Critical Factors—Analytical Hierarchy Process (AHP) for Knowledge-Based and Fuzzy Logic Models for Remote Sensing Data Fusion

There are certain factors that may heavily affect the fire behavior and ignitability. Surface features such as elevation, slope, and aspect may impact the type of ignitions (natural or human-caused), the fire acceleration, and the vulnerability of southern surfaces, respectively (due to increased solar radiation) [36,37]. Regarding the association of land cover and fire hazard, we adopted a differentiated approach taking into account the fire proneness of each land cover type, based on real fire statistics in similar fire regimes like the South European Mediterranean countries [38]. Another important factor constitutes the fire weather. Due to the shortage of climatic data, we used remotely sensed data to determine the condition and the vegetation moisture based on NDVI and NDMI [39,40,41]. Therefore, the higher the NDVI values, the higher the fire hazard due to the abundance of healthy vegetation [42]. In contrast, the higher the NDMI values, the lower the fire hazard due to increased levels of moisture as captured by the ground features. The estimation of anthropogenic impact was determined by establishing distinct zones along the road network (every 100 m) and the inhabited regions (every 200 m) to capture the increased fire activity happening due to road network and Wildland Urban Interface (WUI) proximity [43,44].

Table A1 in the Appendix A presents the knowledge-based weighting of each factor to fire hazard, which reflects the hazard within each class, taking into consideration the local conditions of the island. Table A2 in the Appendix A shows the fuzzification process for each contributing factor based on their inherent characteristics, as previously described. With the term fuzzification, we indicate “the process of converting a crisp input value to a fuzzy value that is performed by the use of the information in the knowledge base” [45].

Besides the determination of an internal weight that interrelates the effect of each factor to forest fire hazard, we assigned an exterior weighting factor to each distinct dimension that reflects the general significance to either forest fire ignition or propagation. To this end, we calculated the influence of each factor through the Analytical Hierarchy Process (a pairwise matrix of factors which measures the relative importance of each factor over the other, conducted with the contribution of experts), as described by Saaty [46]. This process contributes to a more objective weighting ranking, trying to keep consistency between the involved factors. Table 2 provides the calculated weight of each involved factor to fire hazard.

For comparison purposes, we assign each weight to every factor for both methodologies, namely, the AHP-knowledge-based and the AHP-fuzzy logic models. Specifically, the crisp and fuzzy values derived from the Appendix A Table A1 and Table A2 and Table 1 are ranked through the following formula:

Fire hazard per pixel = 0.02 ∗ (Crisp/Fuzzy) Elevation + 0.07 ∗ (Crisp/Fuzzy) Slope + 0.12 ∗ (Crisp/Fuzzy) Aspect + 0.25 ∗ (Crisp/Fuzzy) Land Use + 0.09 ∗ (Crisp/Fuzzy) Distance from roads + 0.04 ∗ (Crisp/Fuzzy) Distance from artificial structures + 0.14 ∗ (Crisp/Fuzzy) NDVI + 0.26 ∗ (Crisp/Fuzzy) NDMI(1)

#### 2.3.2. Comparative Assessment and Validation

In order to keep the most representative and consistent (based on fire history) fire hazard map, a validation approach took place. To this end, the map of the historic burned area for the island of Skiathos was used and overlaid with the fire hazard maps of both techniques. This type of information was retrieved by the spatiotemporal recording of burned areas based on the inter-annual monitoring of Landsat images from 1984 to 2016 [22]. The map with the highest association of fire hazard and burned area will be kept as a prototype for the next stages of analysis. It should be noted that the fire hazard levels have been determined based on equal intervals classification (interval of 0.2 for a scale from 0 to 1) for both methodologies for comparison purposes.

#### 2.3.3. Spatiotemporal Analysis and Spatial Statistics

Three additional procedures took place in order to explore the spatiotemporal dynamics and heterogeneity of fire hazard. Initially, the percentage change of fire hazard between 1996 and 2016 was conducted, so that we can explore any change occurred within this timeframe. Equation (2) describes the percentage change of fire hazard from 1996 to 2016:

(2) $${\mathrm{Fire}\;\mathrm{hazard}}_{1996–2016}=\frac{{\mathrm{Fire}\;\mathrm{hazard}}_{2016}-{\mathrm{Fire}\;\mathrm{hazard}}_{1996}}{{\mathrm{Fire}\;\mathrm{hazard}}_{1996}}\times 100$$

Next, the change of fire hazard levels was computed as an additional index of fire hazard. The class intervals were determined in exactly the same thresholds for both maps in order to allow comparability and fire hazard transition.

Afterwards, spatial statistics techniques were used in order to refine the outcomes and explore the potential change of heterogeneity of fire hazard. Firstly, we performed an Exploratory Spatial Data Analysis (ESDA) to understand the data behavior and trends (i.e., isotropy or anisotropy). One specific part incorporated the application of standard deviation Voronoi map in order to have a first indication of spatial variability.

Following, we had to locate any clustering process that requires special attention, especially in a forest fire event where the fire may propagate easily through the most susceptible regions. This process has been conducted through the exploration of Local Indicators of Spatial Association (LISA), as described by Anselin [47]. This process would allow us to locate the (statistically significant) clusters of high-high (low-low) values and the potential existence of outliers. Next, we will determine any potential spatial autocorrelation of fire hazard across the entire study area through the estimation of Global Moran’s *I* Index.

The local Moran’s *I* index for mapping clusters and outliers is given by the following equation [47,48,49]:

(3) $$M_{i}=\frac{y_{i}-Y_{mean}}{{A_{i}}^{2}}\sum_{j=1}^{m}w_{i,j}\;\left(y_{j}-Y_{mean}\right)$$

where *y_i_* = attribute of *i*, *Y_mean_* = mean of attribute *y*, and *w_i,j_* = spatial weight between *i* and *j*:

(4) $${A_{i}}^{2}=\frac{\sum_{j=1}^{m}{\left(y_{j}-Y_{mean}\right)}^{2}}{m-1}$$

*j* ≠1, *m* = total number of entities.

The global Moran’s *I* index for measuring spatial autocorrelation and pattern type (clustered, dispersed, random) based on the location and attributes of features is given by the following equation [50,51,52,53]:

(5) $$\mathrm{Mi}=\frac{m}{C_{i}}\frac{\sum_{i=1}^{m}\sum_{j=1}^{m}w_{i,j}\;k_{i}\;k_{j}}{\sum_{i=1\;}^{m}{k_{i}}^{\prime 2}}$$

where *k_i_* = the abstraction of entity *i* from the mean (*y_i_*—*Y_mean_*), and *w_i,j_* = spatial weight between *i* and *j*:

(6) $$C_{i}=\sum_{i=1}^{m}\;\sum_{j=1}^{m}w_{i,j}$$

The z-score and *p*-values determine if the spatial processes are statistically significant, in other words, if we can reject the null hypothesis which indicates total randomness of the studied phenomenon. 

Finally, we analyzed the two semi-variograms to conclude the spatial dependence change between those two years. We used the Ordinary Kriging in order to compute the semi-variograms which can be calculated through the following formula [54,55]:

(7) $$\gamma (d)=\frac{1}{2N\left(d\right)}\sum_{i=1}^{N\left(d\right)}{\left[v\left(x_{i}\right)-v\left(x_{i}+d\right)\right]}^{2}$$

where: γ(d) = the semi-variance at distance *d*, *v*(*x_i_*) = the value of the variable *v* at the location of *x_i_*, *d* = lag distance, and *N(d)* = number of sampled pairs separated by *d*.

In order to validate and further explore the parameters of semi-variogram modeling, we used Ordinary Least Squares and Geographically Weighted Regression techniques.

## 3. Results

### 3.1. Fire Hazard Maps of AHP-Knowledge-Based and AHP-Fuzzy Logic Models for 1996 and 2016

**Figure 3:**
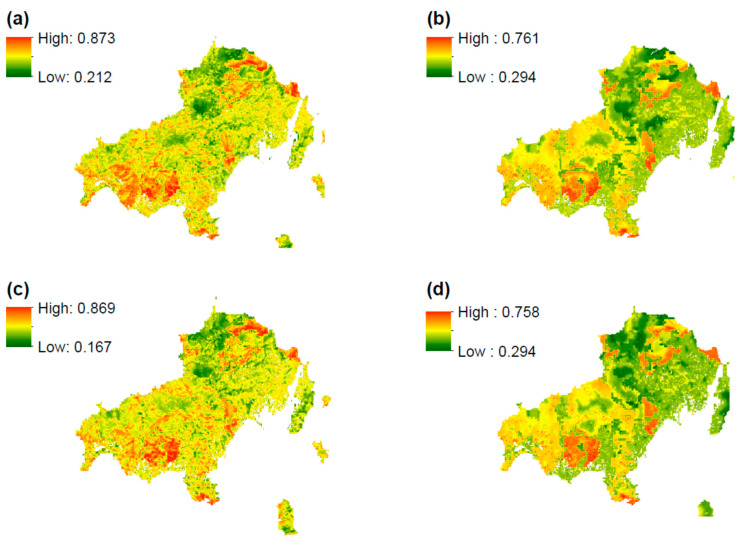
(**a**) AHP-knowledge-based fire hazard map for 1996, (**b**) AHP fuzzy logic fire hazard map for 1996, (**c**) AHP-knowledge-based fire hazard map for 2016, and (**d**) AHP fuzzy logic fire hazard map for 2016.

**Figure A1:**
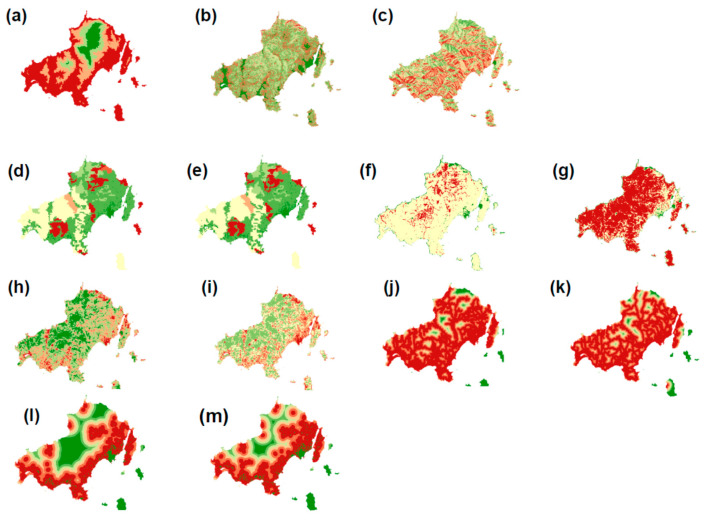
Knowledge-based individual fire hazard maps for 1996 and 2016 (for all involved factors) (**a**) Elevation 1996–2016, (**b**) Slope 1996–2016, (**c**) Aspect 1996–2016, (**d**) Refined land cover 1996, (**e**) Refined land cover 2016, (**f**) NDVI 1996, (**g**) NDVI 2016, (**h**) NDMI 1996, (**i**) NDMI 2016, (**j**) proximity to road network 1996, (**k**) proximity to road network 2016, (**l**) proximity to human residences 1996, and (**m**) proximity to human residences 2016.

**Figure A2:**
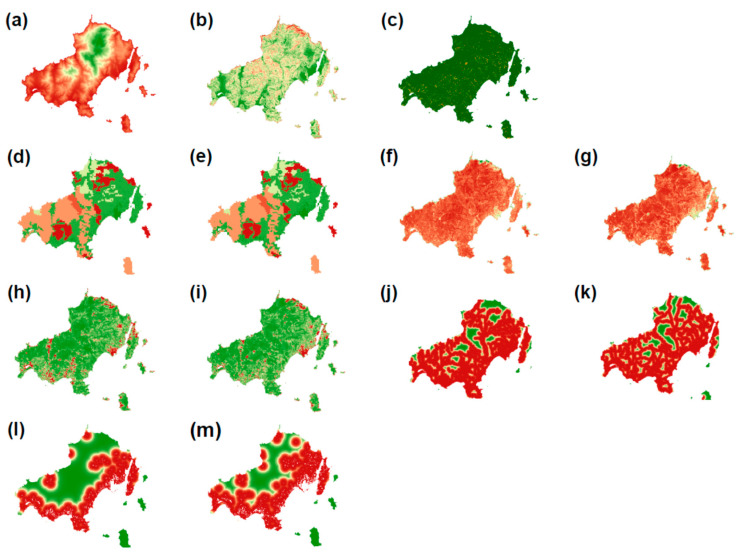
Fuzzy logic individual fire hazard maps for 1996 and 2016 (for all involved factors) (**a**) Elevation 1996–2016, (**b**) Slope 1996–2016, (**c**) Aspect 1996–2016, (**d**) Refined land cover 1996, (**e**) Refined land cover 2016, (**f**) NDVI 1996, (**g**) NDVI 2016, (**h**) NDMI 1996, (**i**) NDMI 2016, (**j**) proximity to road network 1996, (**k**) proximity to road network 2016, (**l**) proximity to human residences 1996, and (**m**) proximity to human residences 2016.

The first section of results summarizes the individual fire hazard of each factor for both models (Appendix A Figure A1 and Figure A2). The integration of AHP in knowledge-based (AHP-KB) and fuzzy logic (fuzzy AHP) modeling produced the final fire hazard maps for both reference years. Here, we may compare/assess the two methodologies, namely, the impact of crisp and fuzzy boundaries for all factors, so that we can keep the most representative one. Figure 3 shows the fire hazard maps for 1996 and 2016 for both techniques. As we can see, the highest fire hazard value of AHP-KB amounts to 0.87 (over 1), whereas the value for the same index of fuzzy AHP is 0.76 for 1996. We have similar results for 2016, where the highest fire hazard value of AHP-KB amounts to 0.86, whereas the value for the same index of fuzzy AHP is 0.75. A reversing trend is observed for the lowest values of fire hazard, where the lowest fire hazard value of AHP-KB is 0.21 compared to 0.29 for the fuzzy AHP map in 1996. In 2016, the respective value of fuzzy AHP is almost double the AHP-KB (0.29 versus 0.16). 

In addition, we conclude that both the fuzzy AHPs present a more uniform map, most probably due to fuzziness of values in contrast to crisp values. Hence, AHP-KB maps tend to present higher variability. However, the phenomenon of spatial variability will be examined in the subsequent sections. Even though the spatial patterns between the two methodologies are alike, there are some distinct differences. The AHP-KB maps are characterized by more extensive high and moderate fire hazard regions compared to fuzzy AHP maps which present larger territories of low and moderate fire hazard. Nevertheless, the selection of the most appropriate methodology and maps will be conducted in the validation approach.

### 3.2. Comparative Assessment and Validation—Selection of the Most Representative Technique

This section explores the validity of each fire hazard map for the two methodologies based on real fire history data. Appendix A Table A3 presents the overlay between real burned area and fire hazard levels. The conclusions of the previous section have been confirmed, indicating that the most susceptible regions (of high and very high hazard) of the AHP-KB map have already been affected by forest fire events. Specifically, 28.6% (1996) and 33.9% (2016) of high and very high hazard have been burned for the AHP-KB maps, whereas the respective percentages for the fuzzy AHP maps amount to 8.3% and 12.7%. The major territory affected by fires has been classified as moderate fire hazard (from 65% to 70% for the AHP-KB maps, and about 85% for the fuzzy AHP maps). A slight percentage of historically burned area has been classified as low hazard for the AHP-KB maps (2.1% and 0.9% for 1996 and 2016, respectively), whereas the fuzzy AHP maps classified these regions as low hazard with greater percentages (6.7% and 2.2% for 1996 and 2016, respectively).

The above results indicate that despite the certain advantages that may have the fuzzy logic modeling alleviating the boundaries of fire hazard for each factor, the AHP-KB modeling was proven more representative in relation to real fire history data. To this end, we selected the AHP-KB maps in order to further examine the spatiotemporal dynamics and variability in a timeframe of 20 years. 

### 3.3. Spatiotemporal Analysis

**Figure 4:**
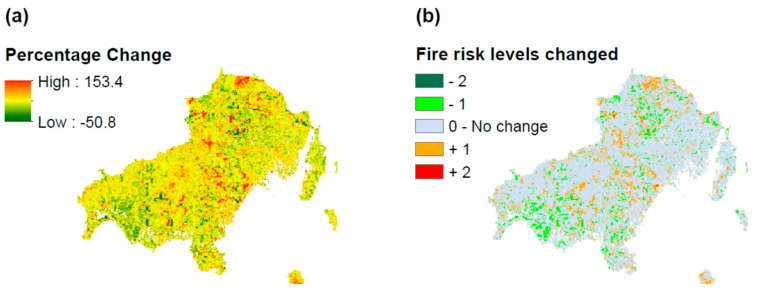
(**a**) Spatiotemporal percentage change of fire hazard, and (**b**) fire hazard levels changed (transition) from 1996 to 2016.

The current section explores the long-term evolution of fire hazard through the determination of spatiotemporal percentage change as well as the transition of fire hazard levels. Figure 4a depicts the spatiotemporal percentage change of fire hazard from 1996 to 2016, where we can observe that the majority of the territory of the island retained the fire hazard status in the examined timeframe. However, some regions in the western, northern, and central parts of the island have significantly increased their fire hazard, potentially due to the interplay of critical factors, such as the NDMI, NDVI, and the presence of more artificial structures. The NDVI evolution clearly shows healthy vegetation status, the NDMI evolution is related with lower levels of moisture, which seems a reasonable evolution under the effect of climate change, and the increase of artificial structures was expected in a quite sophisticated island with increased touristic flows and weak institutional spatial planning. Moreover, some other regions improved their fire hazard status (moving to lower fire hazard), especially in the very western part as well as in sparse areas across the entire island. The highest fire hazard change observed was +153.4%, which means 3 times more vulnerable area in terms of fire hazard, whereas the major reduction of fire hazard was estimated to be 50.8%. 

Figure 4b allowed us to determine the change of fire hazard levels in 20 years. We conclude that most of the territory retained the same fire hazard, whereas there are some parts which increased their fire hazard by one level (in the western, central, and northern parts of the study area). Some other parts decreased their fire hazard by one level and these areas can be located in the central, western, southern, and northern regions. The territories that increased or decreased their fire hazard by two levels are very few and can be located sparsely in the entire study domain.

### 3.4. Geostatistical Analysis

The first necessary step before modeling constitutes the Exploratory Spatial Data Analysis (ESDA) of our data. The histograms of both fire hazard maps indicate a normal distribution. The mean and median value for the fire hazard map of 1996 is 0.53, whereas the standard deviation amounts to 0.09. The mean value for the 2016 fire hazard map is 0.54, whereas the median and standard deviation values amount to 0.53 and 0.08, respectively.

**Figure 5:**
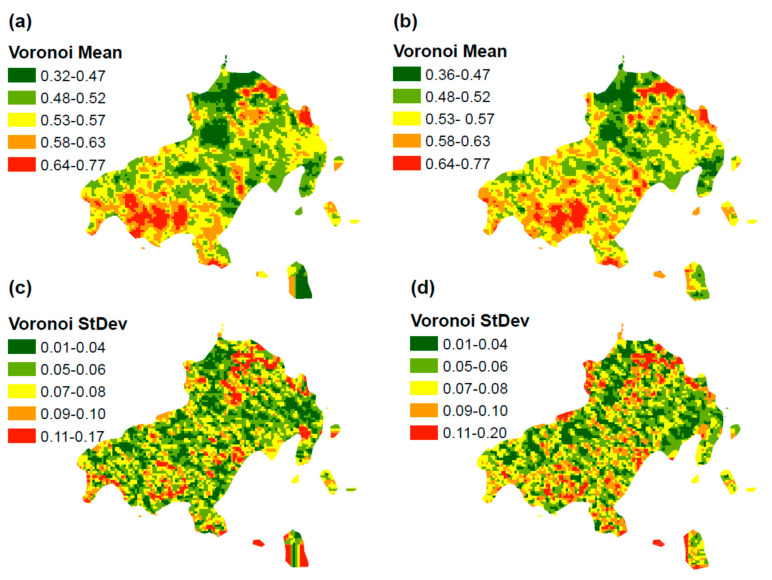
(**a**) Voronoi mean map 1996, (**b**) Voronoi mean map 2016, (**c**) Voronoi standard deviation map 1996, and (**d**) Voronoi standard deviation map 2016.

Next, we used Voronoi mean maps to explore any potential anisotropic pattern. As we can observe from the Figure 5a,b, an isotropic pattern is obvious, since the potential spatial autocorrelation pattern is the same in any direction. Both maps (1996 and 2016) clearly present isotropic behavior. It is apparent for the fire hazard categories, since these categories expand uniformly in any direction. This piece of information is useful for the development of semi-variogram in order to exclude any anisotropic behavior in the process of modeling. Besides, the Voronoi standard deviation maps indicate that both maps (1996 and 2016) seem to manifest a significant degree of local variability (Figure 5c,d), however, further analysis will be conducted in the following section in order to examine the spatial variability in detail. 

**Figure 6:**
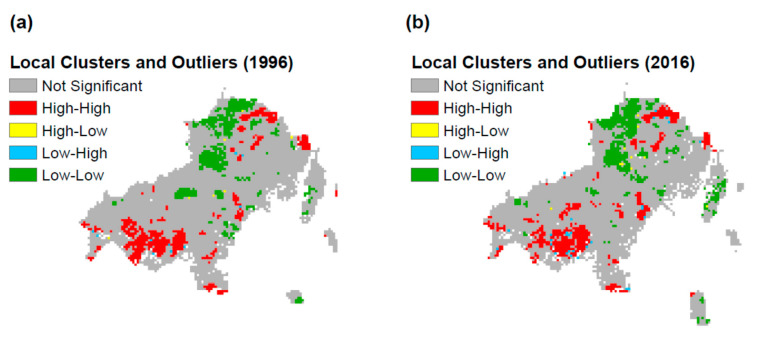
(**a**) Local Indicators of Spatial Association 1996, and (**b**) Local Indicators of Spatial Association 2016.

Following, we explore the potential local and global spatial autocorrelation. As we can observe from Figure 6, there are some clusters of high values especially in the southwestern as well as in the northeastern and eastern parts of the island. The clusters of low values are located in the northern regions of the study area. The cluster process between 1996 and 2016 has not dramatically changed, however, some remarks should be noted. The 2016 cluster of high fire hazard in the southwestern part has been less compact compared to 1996. In addition, it seems to have been expanded in several central and northern parts of the island. On the contrary, the 2016 cluster of low fire hazard has been expanded in the northern and decreased in the central part of the island. Furthermore, there are a few spots of high-low values and vice versa, however, their size is quite limited and primarily located in the borderline between high and low fire hazard zones.

The next step included the exploration of global spatial autocorrelation existence. Therefore, we calculated the global spatial autocorrelation (Moran’s *I*) and concluded that there is a positive spatial autocorrelation promoting the clustering process. The results of Moran’s *I* indicate that there is less than 1% possibility that the clustered spatial pattern could be due to randomness (null hypothesis). The *p*-values for both patterns are 0.01 and the corresponding z-score values are greater than 2.58.

**Figure 7:**
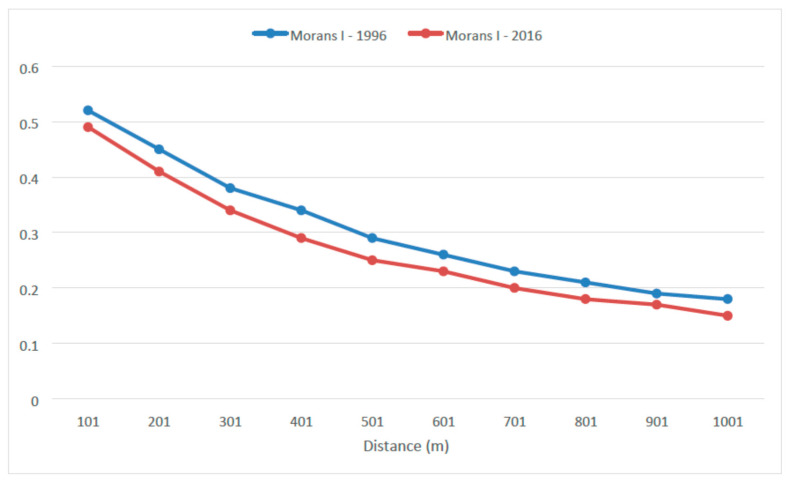
Moran’s *I* index for fire hazard (1996 and 2016) in relation to distance.

Figure 7 shows a similar tendency for both fire hazard maps, where Moran’s *I* values decrease as the distance increases. In the last 1000 m, the Moran’s *I* values for 1996 and 2016 are 0.18 and 0.15, respectively. The Moran’s *I* index for 1996 is always higher than the index for 2016, indicating that the spatial autocorrelation is extended to a greater distance than the corresponding value of 2016.

Finally, we examine the semi-variance modeling for both fire hazard maps. We used the Ordinary Kriging process for estimating the modeling parameters and the Geographically Weighted Regression (GWR) technique in order to examine the appropriateness of model fitness and accuracy of modeling. For the variogram modeling, four models have been adopted, namely, the stable (a somewhat optimized process applied by ArcGIS), Spherical, Exponential, and Gaussian models. The most successful one is considered the stable followed by the Exponential model for both fire hazard maps. The stable models (Table 2) show very high *R*^2^ index (0.96), followed by the Exponential models which present a high *R*^2^ index (0.9) as well. The appropriateness of the stable model is confirmed by the very low Sum of Squared Residuals (RSS) (0.6), whereas the Exponential models present a little higher RSS (1.32 and 1.37 for 1996 and 2016, respectively). Definitely, it should be noted that these values seem so optimized because of the abundancy of the data modeled (every 100 m). In addition, it is remarkable that the major distance (3096 m) for 1996 where spatial autocorrelation exists is by far greater than the respective distance (1626 m) for 2016. Inevitably, the sill values for 1996 are greater than those of 2016. The same trends are observed for the other types of models as well, but with lower degree of appropriateness and accuracy (Table 3).

Here, we should emphasize the fact that we should be very cautious when examining geostatistical data. For instance, when we applied the Ordinary Least Squared (OLS) technique, the *R*^2^ indices ranged in very low levels, except the stable model. However, this is reasonable, indicating the inappropriateness of this method due to the existence of spatial autocorrelation of residuals. The spatial autocorrelation of residuals makes the OLS inappropriate. To deal with this fact, we decided to apply the Geographically Weighted Regression (GWR) technique to validate the appropriateness of model fit. Table 3 summarizes the most critical parameters of semi-variance modeling. 

## 4. Discussion

Forest fires are inherently a complex phenomenon and attract a lot of attention due to the unexpected consequences that they may cause to human and natural environment. Many researchers have estimated the fire hazard/risk integrating natural, anthropogenic [11,14], and meteorological factors [13,56,57]. However, the impact of weather is considered of utmost importance, since fire behavior (and the corresponding degree of impacts) is heavily reliant on weather conditions. The absence of long-term weather data in the study area has been tackled by the adoption of proxy indices, such as the NDVI and NDMI, which may sufficiently and universally describe the state and moisture of vegetation. On the other hand, even though most research is logically based on fire flammability and potential fire behavior of fuels [14,58,59,60], we connected the fuels’ hazard with the probability of a fire event based on real fire statistics (fire susceptibility) [38]. 

Another crucial aspect is the comparative evaluation between AHP-knowledge-based and AHP-fuzzy logic modeling. The issue lies in the type of classification boundaries for each factor. The former is related to crisp boundaries, whereas the latter is leading to “soft” boundaries based on the fuzzy set theory [61]. Despite the fact that each method has its own merits, forest fire researchers have applied these techniques interchangeably, or in an integrated way [10,11,12]. The comparative assessment of the two techniques with historical burned area revealed that AHP-fuzzy logic modeling underestimated the fire hazard compared to AHP-knowledge-based modeling.

Generally, works focusing on long-term spatiotemporal analyses [58] and variability [60,62] of fire hazard are very few. Our work has expanded the analysis in both ways. We simultaneously monitored the spatiotemporal fire hazard change and the potential transformation of high-risk clusters in conjunction with fire hazard heterogeneity in a timeframe of 20 years. Spatial variability/heterogeneity of fire hazard could be a critical information for fire and land use planners, since it is directly related with the ease of transmissibility of forest fires. The combined pieces of analyses could provide targeted preventative measures. It should be highlighted that most of the data has been acquired, transformed, edited, and integrated by multiple and differentiated remotely sensed data sources, without which, the comprehensive analysis of fire hazard would be infeasible. 

However, some limitations of the paper should be handled as future perspectives. One aspect is related with the nature of the so-called dynamic factors. The impact of new road segments, dwellings, and the NDVI index (depicting the state vegetation in the same month, the recognition of burned areas, afforestation, etc.) can be successfully captured in a moment after 20 years. However, the NDMI may have some inter-annual variability that is not captured in one year. It would be a future action to monitor the NDMI indices for each year of the timeframe in order to retain and use average values instead of one-year values. In the same context, a future work could include the analysis of dynamics of the fire hazard clusters along time and space by year from 1996 to 2016. Another aspect is related with the different spatial resolution of the involved data. This fact might provoke loss of critical information, since data of higher resolution might be disappeared (small isolated dwellings, etc.) due to the lower resolution of the final product. However, the data with high spatial resolution (roads, buildings, etc.) has been transformed to extensive regions (extensive zones—buffers along the road and human settlements network), reducing the degree of data loss due to the upscaling process. On the other hand, the very high spatial resolution may lead us to increased cost of data acquisition and processing, whereas the added value of details with extremely small size may be marginal. That is why we defined the size of the final pixel to 30 m. We consider that 30 m resolution is appropriate for a forest fire phenomenon, a resolution which is the same with some of the most critical factors (vegetation status and moisture). However, the adoption of Sentinel images could provide a little higher resolution (i.e., 20 m) that could improve our results. Another perspective is related with the land cover data (CLC). CLC data has a resolution of 100 m. Even though it may be characterized by adequate quality, the spatial resolution should be improved in the future for such vital data. Land use classification of Sentinel-2 images could increase the usability of this type of data.

## 5. Conclusions

Forest fire hazard estimation is a crucial factor for prevention of large and high severity forest fires that could have profound implications on natural and anthropogenic ecosystems. Spatiotemporal analysis of fire hazard may help decision-makers to assimilate the inherent long-term factors that affect fire hazard such as climate change and/or human intervention on natural environment. The knowledge of fire hazard dynamics may provide the fire and land managers with valuable fire prevention tools. Hence, appropriate land management strategies should be adopted, such as the enhancement of spatial planning for greater protection of natural environment from the unregulated urbanization and the rational forest management through the decomposition of dense forests (e.g., thinning, etc.). In addition, more fire-specific measures should be developed, such as the rational planning of firebreaks, the development of efficient watchtowers networks [63], and spatial decision support systems that would allow the finding of best locations for the fire vehicles, aiming at the minimization of travel time for initial attack [64,65]. It should be noted that significant parts of spatial analysis would be impossible to be conducted without the contribution of remote sensing techniques for the acquirement and manipulation of the necessary information.

## Figures and Tables

**Table 1:** Classification of types of data used in forest fire hazard modeling ^1^.

Type of Data	Spatial Resolution	Purpose	Source
Digital Elevation Model	5 m	Generating Elevation, Slope, and Aspect grids	[27]
Land uses 1990	100 m	Flammability characterization	[21]
Land uses 2012	100 m	Flammability characterization	[21]
Road network 1996	Digitized, based on 5 m orthophoto image—Rasterized data of 5 m	Development of zones (buffers) adjacent to road network—Increased vulnerability	Digitization
Road network 2016	Rasterized data of 5 m	Development of zones (buffers) adjacent to road network—Increased vulnerability	[29]
Inhabited regions—Artificial structures 1996	Digitized, based on 5 m orthophoto image—Rasterized data of 5 m	Development of zones (buffers) adjacent to human settlements network—Increased vulnerability (Wildland Urban Interface)	[27]
Inhabited regions—Artificial structures 2016	Digitized, based on Google Earth images Rasterized data of 5 m	Development of zones (buffers) adjacent to human settlements network—Increased vulnerability (Wildland Urban Interface)	Google Earth
NDVI 1996	30 m	Characterization of vegetation health status in relation to fire hazard	USGS
NDVI 2016	30 m	Characterization of vegetation health status in relation to fire hazard	USGS
NDMI 1996	30 m	Vegetation water content—Drought conditions	USGS
NDMI 2016	30 m	Vegetation water content—Drought conditions	USGS

^1^ NDVI: Normalized Difference Vegetation Index, NDMI: Normalized Difference Moisture Index, USGS: United States Geological Survey.

**Table 2:** Weights derived from the Analytical Hierarchy Process (all the stages of the AHP are thoroughly described in the Appendix A).

Factor	Weight
Elevation	0.02
Slope	0.07
Aspect	0.12
Land Uses	0.25
Distance from roads	0.09
Distance from towns	0.04
NDVI	0.14
NDMI	0.26

**Table 3:** Semi-variance modeling parameters for fire inappropriateness models of 1996 and 2016 ^1^.

**Model 1996**	**Nugget**	**Partial Sill**	**Sill**	**Major Range**	***R*^2^ (GWR)**	***R*^2^ (OLS)**	**RSS**	**Sigma**
Stable	0	0.0075	0.0075	3096	0.9661	0.8910	0.6708	0.0127
Spherical	0.0053	0.0018	0.0071	2666	0.8947	0.5170	1.3899	0.0183
Exponential	0.0044	0.0027	0.0072	2437	0.9050	0.5840	1.3225	0.0178
Gaussian	0.0056	0.0015	0.0071	2241	0.8902	0.4864	1.4226	0.0185
**Model 2016**	**Nugget**	**Partial Sill**	**Sill**	**Major Range**	***R*^2^ (GWR)**	***R*^2^ (OLS)**	**RSS**	**Sigma**
Stable	0	0.0071	0.0071	1626	0.9686	0.9181	0.6148	0.0121
Spherical	0.0048	0.0019	0.0068	1741	0.8778	0.5066	1.4379	0.0186
Exponential	0.0035	0.0032	0.0068	1364	0.8979	0.6552	1.3715	0.0181
Gaussian	0.0048	0.0019	0.0068	1122	0.8705	0.4687	1.4874	0.0189

^1^ GWR: Geographically Weighted Regression, OLS: Ordinary Least Squared, RSS: Sum of Squared Residuals.

## Appendix A

**Table A1:** Knowledge-based weighting of fire hazard factors.

**Slope (Degrees) ([10], Adapted)**	**Weight**	**Fire Hazard**	**Aspect ([57,58], Adapted)**	**Weight**	**Fire Hazard**
0–5	1	Very low	Smooth ground	1	Null
5–15	3	Low	North	2	Very low
15–25	5	Moderate	Northeast	3	Low
25–35	7	High	Northwest	4	Lower than mean
35–45	9	Very high	East	5	Moderate
>45	10	Extremely high	Southeast	6	Higher than mean
			West	4	Lower than mean
			Southwest	8	Very high
			South	10	Extremely high
**Elevation (Meters)**	**Weight**	**Fire Hazard**	**Distance from Roads (Meters) ([11], Adapted)**	**Weight**	**Fire Hazard**
0–100	10	Extremely high	400–500	2	Low
100–200	8	Very high	300–400	5	Moderate
200–300	5	Moderate	200–300	7	High
>300	2	Low	100–200	8	Very high
			0–100	10	Extremely high
**Land Uses** **([37,59], Adapted)**	**Weight**	**Fire Hazard**	**Distance from Towns (Meters) ([57,58], Adapted)**	**Weight**	**Fire Hazard**
Airports; Discontinuous urban fabric	1	Very low	800–1000	2	Very low
Land principally occupied by agriculture, with significant areas of natural vegetation; Olive groves; Complex cultivation patterns; Sparsely vegetated areas	3	Low	600–800	3	Low
Broad-leaved forest	5	Moderate	400–600	5	Moderate
Coniferous forest	7	High	200–400	7	High
Mixed forest	8	Very high	0–200	8	Very high
Natural grasslands	9	Very high			
Sclerophyllous vegetation; Transitional woodland-shrub	10	Extremely high			
**NDVI (Values)** **[41]**	**Weight**	**Fire Hazard**	**NDMI (Values)** **[65]**	**Weight**	**Fire Hazard**
−0.33–0.2	0	Null	>0.3	1	Very low
0.2–0.5	5	Moderate	0.15–0.3	4	Moderate
>0.5	7	High	0–0.15	7	High
			−0.22–0	9	Very high

**Table A2:** Fuzzy modeling of fire hazard factors.

Factor	Fuzzification Process (Fuzzy Membership)	Properties *
Elevation	Fuzzy Linear	As the elevation increases the possibility of being a member decreases
Slope	Fuzzy Linear	As the slope increases the possibility of being a member increases
Aspect	Fuzzy Gaussian: Threshold = 180; Spread = 0.01	As aspect deviates from South (the Midpoint) in any direction, the possibility of being a member diminishes
Land Uses	Fuzzy—Division	The fuzzification process involves the division of the categorical value by 10
Distance from roads	Fuzzy Small—Euclidean Distance from roads: Threshold = 200; Spread = 5	The emphasis is given on the area close to roads, especially within 200 m. After this threshold, the possibility of being a member is drastically decreased.
Distance from towns	Fuzzy Small—Euclidean Distance from towns: Threshold = 500; Spread = 5	The emphasis is given on the area close to inhabited regions, especially within 500 m. After this threshold, the possibility of being a member is drastically decreased.
NDVI	Fuzzy Large: Threshold = 0.35; Spread = 0.1	The emphasis is given on the most susceptible regions which include shrubs and pure forests. Below this threshold, the possibility of being a member is drastically decreased.
NDMI	Fuzzy Small: Threshold = 0.2; Spread = 0.1	The emphasis is given on the most susceptible regions which include the driest territory. Above this threshold, the possibility of being a member is drastically decreased.

* Being a member indicates high fire hazard.

**Table A3:** Comparative assessment of fire hazard maps based on real fire history [Hectares (ha) − % of fire hazard for the entire island)].

Ha (% of the Total)	Low	Moderate	High	Very High
Fire hazard 1996 AHP-KB	12.5 (2.1%)	408.6 (69.3%)	166 (28.1%)	3 (0.5%)
Fire hazard 1996 Fuzzy AHP	39.3 (6.7%)	501.9 (85.1%)	49 (8.3%)	0 (0.0%)
Fire hazard 2016 AHP-KB	5 (0.9%)	384.8 (65.2%)	195.8 (33.2%)	3.9 (0.7%)
Fire hazard 2016 Fuzzy AHP	13 (2.2%)	502 (85.1%)	75 (12.7%)	0 (0.0%)

### Analytical Hierarchy Process Stages

The AHP process is based on the consistency of the calculated weighting factors. To this end, the consistency ratio should be less than 0.1, which is given through the following division:

(A1) $$\mathrm{CR}=\frac{\mathrm{CI}}{\mathrm{RI}}$$

where CR = Consistency Ratio, CI = Consistency Index, and RI = Random Consistency Index. The Random Consistency Index is based on the number of involved factors. In our case, this index takes the value of 1.41.

The Consistency Index is calculated through the following formula:

(A2) $$\mathrm{CI}=\frac{\left(\lambda \max-\mathrm{nf}\right)}{\left(\mathrm{nf}-1\right)}$$

where CI = Consistency Index, λmax = is the maximum eigenvector, and nf = number of factors.

The λmax is calculated through the following equation:

(A3) $$\lambda \max=(\frac{1}{\mathrm{nf}})[\left(\sum_{i=1}^{\mathrm{nf}}p_{i}*c_{i})/w_{i}\right]$$

where p_i_ = the pair-wise ranking of each factor, c_i_ = the weighting factor from the pair-wise comparison, and w_i_ = the final weight for each factor.

Based on the above calculations, the λmax = 8.869 and the Consistency Index = 0.124, whereas the Consistency Ratio amounts to 0.088. Hence, the ranking of the involved factors from experts in the field are considered acceptable.
